# Innate Immunity in Systemic Sclerosis Fibrosis: Recent Advances

**DOI:** 10.3389/fimmu.2018.01702

**Published:** 2018-07-23

**Authors:** Paoline Laurent, Vanja Sisirak, Estibaliz Lazaro, Christophe Richez, Pierre Duffau, Patrick Blanco, Marie-Elise Truchetet, Cécile Contin-Bordes

**Affiliations:** ^1^CNRS-UMR 5164, ImmunoConcEpT, Bordeaux University, Bordeaux, France; ^2^Internal Medicine Department, Bordeaux University Hospital, Bordeaux, France; ^3^Rheumatology Department, Bordeaux University Hospital, Bordeaux, France; ^4^Immunology and Immunogenetic Department, Bordeaux University Hospital, Bordeaux, France

**Keywords:** innate immunity, systemic sclerosis, fibrosis, sterile inflammation, future therapeutic

## Abstract

Systemic sclerosis (SSc) is a heterogeneous autoimmune disease characterized by three interconnected hallmarks (i) vasculopathy, (ii) aberrant immune activation, and (iii) fibroblast dysfunction leading to extracellular matrix deposition and fibrosis. Blocking or reversing the fibrotic process associated with this devastating disease is still an unmet clinical need. Although various components of innate immunity, including macrophages and type I interferon, have long been implicated in SSc, the precise mechanisms that regulate the global innate immune contribution to SSc pathogenesis remain poorly understood. Recent studies have identified new innate immune players, such as pathogen-recognition receptors, platelet-derived danger-associated molecular patterns, innate lymphoid cells, and plasmacytoid dendritic cells in the pathophysiology of SSc, including vasculopathy and fibrosis. In this review, we describe the evidence demonstrating the importance of innate immune processes during SSc development with particular emphasis on their role in the initiation of pathology. We also discuss potential therapeutic options to modulate innate immune cells or signaling in SSc that are emerging from these recent advances.

## Introduction

Systemic sclerosis (SSc) is a complex autoimmune disease interconnecting vasculopathy, autoimmunity, and fibrosis features. A large body of evidence has indicated that the adaptive immune system with autoreactive T cells and autoantibodies produced by B cells plays a central role in SSc pathogenesis (1). In addition, inflammatory cytokines produced by the innate immune cells have been detected in the affected tissues of both the early and late stage of SSc, suggesting a role of innate immunity both at the onset and progression of the disease (2–6). This notion was recently reinforced by genomic and genetic approaches that have been undertaken to decipher key and conserved pathophysiological pathways within organs across disease forms (7–9). Apart from genomic approaches, the study of mechanisms governing normal tissue repair has revealed physiological pathways that may be disrupted during SSc as well. The concept of unresolved tissue repair leading to sustained fibrosis has emerged based on a persistent sterile inflammation that converts a self-limited repair response to a non-resolving pathological fibrosis (10, 11). However, the initial events leading to such sterile inflammation remain unclear. Recent data showing that an imbalance in danger-associated molecular pattern (DAMP) release and/or pathogen-recognition receptor (PRR) signaling leads to sustained inflammatory cytokine production by fibroblasts or macrophages may provide the missing link in early events of SSc pathophysiology (11). In addition, plasmacytoid dendritic cell (pDC) activation (12, 13) and type I interferon (IFNα/β, IFN-I) production has also been recently shown to contribute to SSc.

In this review, we focus on recent evidence highlighting the contribution of innate immunity during the course of SSc pathogenesis, primarily at the early stages of disease. We also discuss potential therapeutic options that may modulate innate immune cells or signaling in SSc patients.

## What Can be Learned from Genetic Studies on Innate Immune Function During SSc?

Major technological and analytical advances in the past 10 years have allowed the extraction of critical information from transcriptomic data such as lineage-specific gene expression, networks of interactions, and functional information (14–17). This yielded a novel field of study in the integrated comprehension of SSc pathogenesis, identifying a major contribution of innate immunity.

By analyzing three independent gene expression data sets from SSc skin biopsies, the group of Whitfield proposed interconnected functional modules involved in SSc pathogenesis, two of which involve innate immunity and are dominated by IFN, IFN-inducible genes, and type 2 macrophage (M2) signatures. The three other subnetworks were linked to adaptive immunity, fibrotic processes [response to transforming growth factor beta (TGF-β) and extracellular matrix (ECM) disassembly/wound healing], cell cycle, proliferation, and apoptosis (9). The same group recently identified a common pathogenic signature related to an “innate immune-fibrotic axis” that includes IFN-I and alternatively activated macrophages commonly referred as M2 macrophages and describes new specific pathways and hubs active in the skin and lung (8). Among shared networks, the authors found that the “innate immunity-fibrotic network” is conserved between skin and lung while the internal composition and interactions of gene expression in those tissues vary.

Such large-scale genomic studies paved the way for multiple experimental approaches to determine the molecular processes involved in patients and to establish novel therapeutic options targeting specific organs or shared pathophysiological processes.

## Emerging Concept: SSc as an Over-Repair Pathology

The ability of an organism to efficiently recover from injury whether traumatic, infectious, chemical, or internal is pivotal to maintain its integrity (18). During tissue repair, innate immune cell plasticity actively contributes to the development of an abnormal microenvironment, leading to a shift in the balance between the pro-inflammatory and pro-reparative sides of tissue repair, as recently reviewed (10).

Early SSc is characterized by a perivascular leukocyte infiltrate mainly composed of macrophages and T lymphocytes, reminiscent of the process induced during normal wound healing (19, 20). Whereas normal wound healing is accompanied by a remodeling or resolving stage, abnormal wound healing with chronic activation of immune cells such as macrophages or stromal cells like myofibroblasts fails to resolve fibrosis during SSc. Hence, SSc, specifically diffuse cutaneous forms of the disease, could be considered as a general form of over-repair. The initial trigger of the injury is still unknown, but several lines of recent evidence have brought new hypotheses on its nature.

### Role of Sterile Inflammation in Unresolving Tissue Fibrosis During Scleroderma: Importance of DAMP/PRR Imbalance

Recognition of pathogen-associated molecular patterns (PAMPs) or endogenous DAMPs by innate immune cells as well as non-immune cells is the first line of response to pathogen or sterile tissue injury. DAMPs, mainly produced by epithelial cells, are heterogeneous in form encompassing early produced and highly diffusible Ca^2+^, H_2_O_2_, reactive oxygen species (ROS), adenosine tri-phosphate, self-nucleic acids, but also proteins like high-mobility group protein 1, heat shock protein, S100 proteins, and fragments of the ECM. The recognition of PAMPs and DAMPs relies on cell surface, endosomal, and cytosolic PRRs that include toll-like receptors (TLRs), Nod-like receptor, Rig-I-like receptors (RLRs), cyclic GMP-AMP synthase, and receptor for advanced glycation end products. Innate immune signaling triggered by DAMPs during sterile inflammation or the persistence of pathogens such as endogenous viruses might represent an important pathway responsible for converting self-limited regenerative repair into an unresolved fibrotic process during SSc. Hence, innate immune signaling *via* TLRs was recently proposed as a key driver of persistent fibrotic response in SSc and other fibrotic-related diseases (11).

Overexpression of TLR4 and its two co-receptors CD14 and myeloid differentiation factor 2 (MD-2) has been described in SSc-affected skin and lung. TLR4 expression was mainly associated with macrophages, fibroblasts, and myofibroblasts (21). In the skin, TLR4 expression correlated to fibrosis severity measured by modified Rodnan skin score. *In vivo*, chronic TLR4 activation leads to sustained nuclear factor kappa-light-chain-enhancer of activated B cells (NFκB) signaling, resulting in macrophage activation and a profibrotic profile (22). Work from the Varga lab recently demonstrated that endogenous DAMP activation of TLR4 can contribute to converting self-limited tissue repair responses into uncontrolled ECM deposition during SSc [for recent review, see Ref. (11)]. They proposed that fibronectin, containing alternatively spliced exons encoding type III repeat extra domain (EDA), and tenascin-C are constitutively produced by SSc fibroblasts leading to their accumulation in the skin but also in the blood. Together, fibronectin-EDA and tenascin-C act as strong profibrotic factors during SSc by binding to fibroblasts TLR4, leading to enhanced production of collagen and alpha-smooth muscle actin (α-SMA) expression (23, 24). Deletion of EDA or tenascin-C or disruption of TLR4 signaling resulted in reduced fibrotic response in a murine model of SSc. Furthermore, tensional forces generated within a rigid fibrotic microenvironment were reported to favor exposure of the EDA domain of fibronectin (25), suggesting that increased stiffness of the matrix in fibrotic tissue could favor the bioavailability and profibrotic activity of fibronectin-EDA.

Altered expression of multiple DAMPs/TLRs beyond TLR4 has been described during SSc. Indeed, increased expression of TLR9 was found in human SSc skin biopsies in both early and late stages of the disease and was mainly associated with α-SMA-positive myofibroblasts (26), and a TLR9 signature was detected in SSc skin. *In vitro* treatment of normal cutaneous fibroblasts with the TLR9 ligand unmethylated-CpG-oligodeoxynucleotides (CpG ODN) induced a profibrotic profile involving autocrine TGF-β production. Collectively, these results support the role of TLR9 signaling in SSc. Furthermore, expression of TLR2 (27) and TLR3 (28) is also increased in SSc skin fibroblasts. TLR2 was shown to respond to the endogenous ligand amyloid A, resulting in NFκB activation and increased interleukin (IL)-6 secretion causing inflammation (27). However, the role of TLR3 in SSc pathogenesis remains controversial. TLR3 activation by polyinosinic:polycytidylic acid (poly I:C) stimulates IFN-I production by fibroblasts, which in turn reduces their ability to produce ECM components (28). Conversely, such stimulation was shown to promote the expression of TGF-β by fibroblasts thus contributing to the overall fibrosis (29).

In addition to TLRs, other PRRs have been described to play a role in SSc pathogenesis. The IFN-I stimulatory property of poly I:C on SSc patient fibroblasts was shown not only to rely on TLR3 but also on intracellular RLRs (28). The inflammasome, specifically the NLRP3-inflammasome, was shown to contribute SSc pathogenesis *in vivo* (30) through the induction of the microRNA miR-155, which in turn favors excessive ECM production by fibroblasts, exacerbating SSc (31).

Studies on the contribution of TLR signaling to fibrosis in SSc as well as other fibrotic diseases have generated conflicting results (22, 26, 32–35), suggesting that whether TLR activation leads to pro- or anti-fibrotic effects depends on many factors. The nature of the stimulation (chronic vs acute), of the responding cells (immune or non-immune cells), as well as disease stage (inflammatory vs remodeling) might modulate the effects of TLRs in the fibrotic process. Profibrotic effects of TLR activation seem related to fibroblast and macrophage activation in the context of chronic stimulation, whereas epithelial and other immune cell activation in the context of acute stimulation might lead to anti-fibrotic effects. Although additional PRRs have recently been implicated in SSc, further studies are required to identify their endogenous ligands and mechanisms leading to disease. Nevertheless, PRRs and their signaling pathways may represent multiple novel therapeutic targets in SSc.

### Old Players, New Pathways: Type-2 Macrophages, Platelets, and Mastocytes

Macrophages and platelets have emerged as key players not only during tissue homeostasis and repair but also fibrosis, recently reviewed in Ref. (36, 37).

We and others have defined the profibrotic role of platelets in SSc. The Distler group has shown that serotonin [5-hydroxytryptamine (5-HT)] stored in platelets strongly induces ECM synthesis in interstitial fibroblasts *via* activation of 5-HT_2B_ receptors (5-HT_2B_) in a TGF-β-dependent manner (38). Our group discovered a pathophysiological loop active in SSc that links vasculopathy and fibrosis. Indeed, we showed that platelet activation induced the production of thymic stromal lymphopoietin (TSLP) by dermal microvascular endothelial cells in an IL-1β-dependent manner. TSLP was found to be strongly expressed in SSc skin endothelial cells and correlated to the severity of skin fibrosis. *In vitro*, TSLP was able to induce a profibrotic profile in both normal and SSc fibroblasts (39, 40).

Infiltration of macrophages in the early skin lesions of SSc patients, particularly in perivascular areas, was first detected over 20 years ago and could lead to secondary activation of adaptative system (19, 20). Since then, numerous studies have established the involvement of macrophages in SSc pathogenesis, notably their alternatively activated counterpart called M2 macrophages as reviewed in Ref. (41). Soluble CD163, a putative marker of M2 macrophages, was shown to be elevated in SSc patients’ blood and associated with their poor clinical outcome (42). These observations have been reinforced by the recent genetic studies showing a prominent M2 macrophage signature in SSc-affected skin and lung (8). However, the activation of lung macrophages in SSc patients with pulmonary fibrosis is distinct from that observed in SSc skin. Activated lung-resident macrophages display a specific increase in the expression of genes related to lipid and cholesterol trafficking, suggesting a switch in their metabolism. Thus, while M2 macrophages are central to the fibrotic process both in skin and lung during SSc, distinct stimuli derived from the organ-specific microenvironment might differentially shape the plasticity of macrophages. In the recent FASSCINATE trial, molecular profiling of skin biopsies revealed that IL-6 receptor blockade by tocilizumab resulted in a reduced M2 macrophage signature observed in SSc skin (43). Accordingly, the blockade of cAMP-specific phosphodiesterase-4, which inhibits differentiation of M2 macrophages as well as IL-6 production, led to an amelioration of fibrosis in a murine model of SSc induced by bleomycin treatment (44). The same group demonstrated that nintedanib, a tyrosine kinase inhibitor targeting vascular endothelial-, fibroblast-, and platelet-derived growth factor receptors, effectively blocked myofibroblast differentiation and reduced pulmonary, dermal, and myocardial fibrosis in transgenic Fra2 mice. This effect was primarily mediated by preventing M2 macrophage accumulation in the affected tissues (45). However, the mechanisms leading to aberrant M2 macrophage polarization and the precise pathways through which M2 macrophages contribute to tissue fibrosis remain unclear. One elegant study by Eming et al. provided novel mechanistic insight to the role of M2 macrophages in fibrosis. Using a murine model of wound healing, IL-4Ra activation by IL-4 and IL-13 was demonstrated to induce the production of resting like molecule alpha by M2 macrophages, which in turn stimulates the production of enzyme lysyl-hydroxylase-2 (LH-2) ultimately contributing to persistent profibrotic collagen cross-linking in fibroblasts (46). This process was shown to be critical for transformation of the tissue into a persistent scar. In humans, Relm-β induces LH-2 in fibroblasts, and expression of both factors was reported to be increased in lipodermatosclerosis, a condition associated with excessive skin fibrosis. Whether this process contributes to SSc is still unknown. The fine mapping of specific macrophage subsets across tissues and during the course of disease, as well as elucidating of the molecular mechanisms underlying macrophages-induced abnormal resolution, will pave the road to the development of new drugs that prevent/limit fibrosis.

Studies in patients and animal models of SSc have demonstrated that mast cells infiltrate the fibrotic skin (47, 48). This infiltration was associated with more severe disease phenotypes (48), but the function and net contribution of mast cells to fibrosis is only beginning to be understood. Mast cells have been suggested to be an important source of TGF-β and thus contribute to the overall fibrosis (49). Furthermore, a recent report using transgenic mice that develop spontaneous skin fibrosis showed a major role for mast cells specifically in inducing inflammation of the skin and the production of ECM and α-SMA by fibroblasts (50). Together with recent observations showing that mast cell deletion ameliorates experimental SSc *in vivo* (47, 51), these results indicate that mast cell targeting in SSc patients may represent an effective therapeutic approach.

Finally, other innate immune players such as natural killer (NK) cells (52, 53) and neutrophils (54) were shown to display altered properties and phenotypes in the blood of SSc patients. However, further studies are required to evaluate the role of NK cells and neutrophils in the SSc pathogenesis, especially in the settings of murine experimental models.

## New Kids on the Block: pDC and Innate Lymphoid Cell (ILC)

Plasmacytoid dendritic cells are innate immune cells specialized in the production of copious amounts of IFN-I (55), and thus play a key role in the initiation of antiviral immune responses (56, 57). IFN-I production by pDCs requires recognition of viral nucleic acids by TLR7 and TLR9, respectively (56, 57). pDCs were also shown to produce IFN-I in response to self-nucleic acids and consequently contribute to the development of multiple inflammatory and autoimmune disorders (58–62). An IFN-I signature, reflected by increased expression of numerous IFN-I-stimulated genes has been reported in patients with SSc (12). Furthermore, genome-wide association studies in SSc have identified polymorphisms in genes involved in the regulation of IFN-I expression in pDCs, particularly IFN-regulatory factor (IRF)-5, IRF-7, and IRF-8 (12). Approximately half of SSc patients (~50%) display an IFN-I signature within their peripheral blood mononuclear cells (63–65) and in fibrotic skin (66). The association between IFN-I signature and SSc disease activity remains controversial as no major impact of the IFN-I signature on pathological features of SSc, including extent of skin fibrosis, autoantibody specificities, and interstitial lung disease, has been reported (63, 65). However, when the profile of IFN-induced chemokines was specifically analyzed in a large cohort of SSc patients, an association was then identified with more severe SSc (67). As pDCs are an important source of IFN-I, numerous groups have investigated their role in SSc. pDCs were indeed detected in the affected skin of SSc patients (65, 68) as well as in the fibrotic skin of mice after bleomycin treatment (13). Furthermore, mice lacking fibrillin-1 (*Fbn1*), which spontaneously develop a stiff skin syndrome that recapitulates the skin fibrosis observed in SSc patients, show a high infiltration of pDCs in the affected skin (69). The frequency of pDCs is reduced in the circulation of SSc patients, likely due to their preferential recruitment into the fibrotic skin (13). Anti-topoisomerase I and anti-nuclear autoantibodies in SSc patients were shown to form immune complexes with apoptotic cell-derived constituents *in vitro* and consequently stimulate IFN-I production by pDCs (70, 71) upon uptake *via* FcγRII and the stimulation of TLR7/9 (70, 71). While such “interferongenic” properties of immune complexes may contribute to the aberrant IFN-I production, an IFN-I signature was not associated with the production of specific autoantibodies detected in the sera of SSc patients (70), suggesting that additional factors may contribute to pDC activation *in vivo*. Furthermore, pDCs in the peripheral blood or fibrotic skin of SSc patients spontaneously secrete CXC motif ligand (CXCL)-4 and IFNα (13, 68). High levels of CXCL4 in the circulation of SSc patients were associated with disease severity including skin fibrosis and pulmonary arterial hypertension (68). CXCL4 was described to potentiate pDC ability to produce IFN-I *in vitro* largely in response to TLR9 stimulation. In addition, CXCL4 was shown to induce both the expression of TLR8 and the ability to produce IFN-I in response to its specific ligands in pDCs (13). Recently, the pathogenic role of TLR8 was confirmed *in vivo* using transgenic mice that express human TLR8 and develop exacerbated skin fibrosis after bleomycin treatment compared with control animals (13). However, whether such exacerbation of disease in TLR8 transgenic animals is dependent on pDCs remains unknown, and the association between CXCL4 levels and the IFN-I signature in SSc patients has not yet been characterized. Ah Kioon et al. showed that bleomycin-induced skin fibrosis is strongly attenuated after selective pDC depletion (13). Furthermore, this model of fibrosis was associated with an IFN-I signature and increased expression of CXCL4 in the affected skin, and pDC depletion significantly reduced the occurrence of these parameters. From a therapeutic standpoint, pDC depletion ameliorated established bleomycin-induced skin fibrosis, indicating that pDCs are critical even in the maintenance of skin fibrosis. This constitutes the first study showing the deleterious impact of pDCs on SSc development *in vivo* (13). Overall, pDCs play a critical role in SSc pathogenesis; however, the molecular mechanisms through which they contribute to the disease require further investigation. This recent progress nevertheless positions SSc as another autoimmune pathology that may benefit from therapeutic targeting of pDCs using depleting or inhibitory antibodies (72).

Innate lymphoid cells were recently described as novel components of the immune system that may be considered as innate counterparts of polarized T helper cells (73). Nevertheless, knowledge on the role of ILCs in SSc remains limited. Wohlfahrt and colleagues have shown elevated numbers of ILC2 in both the peripheral blood and the affected skin of patients with SSc compared with healthy individuals, and their number correlated with the extent of cutaneous fibrosis (74). However, the increased frequency of ILC2 in SSc peripheral blood was not observed in a different study, which instead reported an elevated frequency of CD4 + ILC1 and NKp44 + ILC3 (75).

Nevertheless, in animal models of lung fibrosis induced by bleomycin, IL-33, an alarmin that has been reported to be elevated in SSc patients (76), induced the expansion of ILC2s producing the profibrotic cytokine IL-13 (77). Hence, further investigations are warranted to determine the role of ILC2 in the development of SSc fibrosis.

## Future Directions and Therapeutic Avenues

Significant progress has recently been made in understanding the contribution of innate immunity to SSc fibrosis. Although the precise molecular mechanisms of their action must be further defined, promising new therapeutic targets for SSc have already emerged. Such strategies include blockade of TLR4/MD-2, TLR9, or downstream signaling molecules to limit chronic sterile inflammation, modulation of macrophage polarization to promote resolution and matrix remodeling, and targeting pDCs/IFN-α. This therapeutic challenge is ongoing with many attractive new therapeutic candidates, some of which are currently being tested in Phase III clinical trials (Tables 1A,B). Both the evaluation of potential side effects and identification of biomarkers of patients who would benefit from such therapies are warranted in order to maximize the efficacy of treatment.

**Table 1:** Potential therapeutics and therapeutics in latest clinical trials specific to innate immunity and fibrosis in SSc.

Innate immunity targeted physiopathological pathways	Target	Molecules	Drug name/trade name	Clinical trial in SSc	Primary end-point	Result
**(A) Chronic sterile inflammation**						
	*TLR4/MD-2 inhibition*	Selective TLR4 inhibitor, lipid A mimetic	E5564/Eritoran	None for SSc, tested in sepsis (lack of efficiency)		

		Anti-TLR4	NI-0101			

		Selective TLR4 inhibitor, small molecule	T5342126	None		

	*TLR4/MD-2 inhibition of DAMP*	Tenascin-C A1 domain specific blocking antibody	F16	None		

		Fibronectin-EDA specific blocking antibody	F8	None		

	*TLR4 downstream signaling*	Small molecule binding the Cys747 of the intracellular domain of TLR4	TAK-242	None for SSc, tested in sepsis (lack of efficiency)		

	*TLR7/8/9*	Small molecule or oligonucleotides	CpG-52364, DV-1179, IMO 3100, IMO-8400	None		

	*NFκB*	PDE4 inhibitor	Crisaborole/Eucrisa	None for SSc but Pilot Study Evaluating the Efficacy of a Topical PDE4 Inhibitor for Morphea NCT03351114	Change in dermal thickness of sentinel plaque from Baseline to 12 weeks	

	*pDC*	Anti-BICD2 antibody	BIIB059	None		

	*Type 1 IFN*	Type 1 interferon receptor sub-unit 1 blocking antibody	MEDI-546	Phase I open-label study in diffuse cutaneous SSc NCT00930683	Safety and tolerability of single or multiple intravenous doses	Decreased type I IFN gene expression in whole blood and skin for subjects with positive scores at baseline

**(B) Abnormal resolution**						
	*Fibroblasts*	Selective CB2 agonist	JBT-101/Lenabasum	Phase II + open-labeled extension	Safety and reduction of the mRSS score	Reduction of 8.4 points in the mRSS score in the open-label extension

		Selective CB2 agonist	JBT-101/Lenabasum	Phase III RESOLVE-1 trial NCT03398837	Change from baseline in mRSS	Expected results in 2020

	*Type-2 macrophages*	Anti-IL-6 receptor alpha blocking antibody	Tocilizumab/Roactemra	Phase II FASSCINATE trial NCT01532869	Safety and difference in mean change from baseline in mRSS at week 24	Primary end-point not reached but diminished type-2 signature in the treated arm

		Tyrosine kinase inhibitor	Nintedanib	Phase III SENSCIS trial NCT02597933	Efficacy and safety in SSc patients with interstitial lung disease at week 52	

		PDE4 inhibitor	Crisaborole/Eucrisa	No clinical trial in SSc, but pilot study evaluating the efficacy of a topical PDE4 inhibitor for morphea NCT03351114	Change in dermal thickness of sentinel plaque from Baseline to 12 weeks	

	*TGF-β*	TGF-β isoforms 1, 2, and 3 blocking antibody	Fresolimumab	Phase I open-label trial NCT01284322	Safety and efficacy (molecular assessment of TGF-β responsive genes and improvement in the mRSS)	Inhibition of TGF-β-regulated gene expression and improvement in the mRSS in the fresolimumab treated group

		Soluble guanylate cyclase activator blocking TGF-β-induced release of ECM components from fibroblasts	BAY63-2521/Riociguat	Phase II RISE-SSc trial NCT02283762	Safety and efficacy (change in mRSS at week 52) in patients with diffuse cutaneous SSc	

*BDCA-2, blood dendritic cell antigen 2; DAMP, danger-associated molecular pattern; IFN, interferon; mRSS, modified Rodnan skin score; SSc, systemic sclerosis; TGF-β, transforming growth factor beta; TLR, toll-like receptor; PDE4, phosphodiesterase-4; ECM, extracellular matrix; pDC, plasmacytoid dendritic cell; MD-2, myeloid differentiation factor 2; CB2, cannabinoid receptor type 2*.

## Author Contributions

All authors listed have made a substantial, direct, and intellectual contribution to the work and approved it for publication.

## Conflict of Interest Statement

The authors declare that this work was conducted in the absence of any commercial or financial relationships that could be construed as a potential conflict of interest.
